# Lightning and deep convective activities over the Tibetan Plateau

**DOI:** 10.1093/nsr/nwz182

**Published:** 2019-11-13

**Authors:** Dong Zheng, Yijun Zhang, Yang Zhang, Wen Yao, Fei Wang, Weitao Lyu, Qing Meng, Xiangpeng Fan, Ying Ma, Jun Yang

**Affiliations:** 1 State Key Laboratory of Severe Weather/Laboratory of Lightning Physics and Protection Engineering, Chinese Academy of Meteorological Sciences, China; 2 Department of Atmospheric and Oceanic Sciences/Institute of Atmospheric Sciences, Fudan University, China

Convections over the Tibetan Plateau play a crucial role in the energy and material exchange between the troposphere and stratosphere, impacting the rain-bearing synoptic systems over eastern China. Lightning can be used as a key indicator of strong convection and therefore its characteristics and relationship with convection over the plateau are of great interest to researchers.

The panorama of lightning activity over the plateau has been revealed by recent studies using the optical observation data from the satellite. High-density lightning is located near Naqu and northeastern Qinghai, where the flash densities can be above 5 fl km^−2^ yr^−1^. About 95% of the flashes occur during May to September, with a peak in July in most regions of the plateau. The ratio of intra-cloud lightning to cloud-to-ground (CG) lightning in the mid-southern plateau is approximately 4 [1]. The lightning over the plateau is typically of shorter duration, smaller horizontal extent and weaker optical radiance than that over inland eastern China, with the ratios of median values for these regions being 0.69, 0.88 and 0.58, respectively [2]. The CG lightning over the plateau tends to feature a long-lasting preliminary breakdown process.

The understanding of deep convective systems (DCSs) over the plateau is growing. The DCSs (with 20-dBZ echo tops exceeding 14 km) over the Tibetan Plateau are more frequent than those over central-eastern China and predominantly occur during the period from noon through evening, but are of smaller horizontal scale and weaker convective intensity [3]. A recent investigation based on ground-based radar observations reveals that the regions of DCSs with radar reflectivity larger than 20 dBZ have a mean area of about 120 km^2^ around the Naqu region, with mean 20 and 40-dBZ echo tops of approximately 15.8 and 9.9 km, respectively. The corresponding values for intense DCSs (IDCSs, with 40-dBZ echo tops exceeding 10 km) are approximately 196 km^2^, 16.5 km and 11.3 km, respectively.

Knowledge of plateau thunderstorms is improving. Throughout most of the Tibetan Plateau, the average area and height of precipitation clouds with lightning are an order of magnitude larger and 2–4 km higher than those without lightning, respectively. On average, the thunderstorms over the eastern plateau tend to have larger horizontal extent and those over the south-by-west and southeastern parts of the plateau tend to have higher top. The plateau thunderstorm tends to have a larger-than-usual lower positive charge center (LLPCC) and therefore yield more-frequency inverted intra-cloud flashes in the lower negative dipole [4]. The LLPCC is suggested to be associated with the weak convection and low freezing level of the plateau, and inductive and non-inductive electrifications commonly play important roles [5].

In summary, the outstanding achievements have revealed the law of plateau lightning activity and discovered the uniqueness of the DCS/thunderstorm structures, charge structure and lightning discharge in the plateau relative to other regions. These findings have enhanced our knowledge of the lightning activities in the plateau and provided important information for the study of convection activities in the plateau and their effects on weather and climate.
